# PW03-017 – Combination TNF and IL-1 blockade in PAPA syndrome

**DOI:** 10.1186/1546-0096-11-S1-A243

**Published:** 2013-11-08

**Authors:** P Hoffmann, AK Ombrello, DL Stone, KS Barron, DL Kastner

**Affiliations:** 1 NHGRI, National Institutes of Health, Bethesda, USA; 2 NIAID, National Institutes of Health, Bethesda, USA

## Introduction

The dominantly inherited PAPA syndrome is caused by mutations in *PSTPIP1*. It is one of the least understood monogenic autoinflammatory diseases, both from a pathogenic and treatment perspective. Patients often have persistent symptoms refractory to biologic therapies that necessitate prolonged exposure to high dose corticosteroids. Prior studies have reported increased production of IL-1b and TNF-α by peripheral blood leukocytes. There are Case reports documenting the efficacy of both IL-1 and TNF-α antagonists in treating PAPA but, to date, there are no reports of combination anti-TNF and anti-IL-1 use.

## Objectives

To assess the safety and efficacy of combination TNF-α and IL-1b agents in four patients with refractory PAPA.

## Methods

We studied 4 patients; 3 adults (ages 52, 26, 18) and one pediatric (age 16) patient. Two adults have the A230T mutation and one has the E250Q mutation. The fourth has a novel E257K mutation. Clinically, all 4 patients have pyoderma gangrenosum (PG) lesions and 3 of the 4 have arthritis. All 4 patients failed monotherapy with anti-IL-1 and anti-TNF agents. Initial single-agent treatments and dose ranges were: infliximab 5-10 mg/kg every 4-8 weeks, adalimumab 20-80 mg every 7-14 days, etanercept 25-50 mg every 7 days, and anakinra 100-500 mg daily. Three out of 4 patients required oral prednisone (15-60 mg daily) and IV methylprednisolone in addition to a biologic. Due to ineffective control of symptoms on single-agent treatment, the combination of an anti-TNF and an anti-IL-1 agent was introduced in all 4 patients. Patients with the A230T mutation received infliximab and anakinra and patients with E250Q and E257K received golimumab and anakinra.

## Results

Four patients with PAPA syndrome were treated with the combination of anti-TNF and anti-IL-1 agents. In the 3 patients that required prednisone, after initiation of combination treatment, the dose was successfully decreased to 18-20 mg daily without requiring methylprednisolone boluses. Clinically, there was a significant decrease in frequency and severity of PG lesions and arthritis. Inflammatory markers on single biologic agents were ESR 57-86, CRP 29.5-140 mg/L and on combination treatment were ESR 2-40, CRP 0.62-14.1 mg/L. We did not observe an increase in the frequency of serious infections on combined biologics.

## Conclusion

In patients with PAPA syndrome refractory to a single biologic agent, the combination of an anti-TNF and an anti-IL-1 agent is an effective alternative compared to escalating corticosteroids. Although careful monitoring is essential, in our patients, combination biologic therapy has not been associated with an increase in infections or other adverse events.

## Disclosure of interest

None declared

